# Septic arthritis of the knee: Presentation of a novel irrigation-suction system tested in a cadaver study

**DOI:** 10.1186/1471-2474-12-180

**Published:** 2011-08-07

**Authors:** Atesch Ateschrang, Dirk Albrecht, Steffen Schröter, Bernhard Hirt, Kuno Weise, Jürgen H Dolderer

**Affiliations:** 1 Department of Traumatology and Reconstructive Surgery, BG-Trauma and Medical Centre Tübingen, Eberhard Karls University Tübingen, Schnarrenbergstr. 95, 72076 Tübingen, Germany; 2 Institute for Anatomy, Department for Clinical Anatomy, Eberhard Karls University, Tübingen, Elfriede-Aulhorn-Straße 8, 72076 Tübingen, Germany; 3 Department of Plastic, Hand and Reconstructive Surgery, BG-Trauma and Burn Centre Tübingen, Eberhard Karls University Tübingen, Schnarrenbergstr. 95, 72076 Tübingen, Germany

## Abstract

**Background:**

The established treatment for bacterial arthritis of the knee joint is arthroscopic surgery with irrigation and debridement. The aim of this article is to summarize the relevant data in treating bacterial arthritis of the knee joint, and based on these findings to present a novel irrigation suction system, tested in a cadaver study, as an additional tool in the postoperative treatment phase of arthroscopic surgery for knee joint infections.

**Method:**

The novel automated irrigation-suction system presented here was compared to conventional continuous suction irrigation in a total of six knee joints. All knee joints were filled with 80 ml methylene blue stain and rinsed by two different methods. Fluid specimens were taken after ten and twenty minutes to be compared by photometric extinction measurement at a wave length of 500 nm.

**Results:**

After ten minutes, the average extinction was e_1C _= 0.8 for the continuous suction irrigation and e_1N _= 0.4 for the novel irrigation-suction system. After twenty minutes, we recorded an average extinction of e_2C _= 0.3 for continuous suction irrigation and e_2N _= 0.001 for the novel irrigation-suction system. The students *t-*test revealed superior results after ten and twenty minutes of washing out the knee joints with a p < 0.001 for the novel irrigation-suction system.

**Conclusion:**

A novel irrigation-suction system may be an effective tool for postoperative knee joint irrigation in arthroscopic therapy for bacterial arthritis of the knee. Further animal studies are needed to verify the effects in vivo.

## Background

Septic arthritis is a serious problem, with the knee the most frequently involved joint in adults [1-3]. Clinical outcome not only depends on the number and type of the agent involved and the general state of the patient, but also on the speed and decisiveness with which the diagnosis is made. Previous studies made clear that immediate treatment is essential to avoid unsatisfactory results [1,4-7]. While the established treatment has changed over the last few decades, the old principle 'ubi pus, ibi evacua' has maintained its relevance. Accepted therapy includes joint decompression with mechanical irrigation reducing the amount of infecting organisms, fibrin coatings and necrotic cell detritus combined with the use of antibiotics to produce full functional recovery [3-7]. Primary open arthrotomy with early subtotal synovectomy and immobilisation of the knee [7-9] has been replaced by arthroscopic surgery and early passive motion in treating bacterial arthritis [3-6].

The aim of this article is to summarise the accepted therapy of bacterial knee joint infections, and to present a novel irrigation-suction system that was tested in a cadaver study to be used in the postoperative treatment phase of arthroscopic joint surgery. To our knowledge there is as yet no publication on this topic with the stated aim of developing a novel automatic irrigation-suction system as an additional tool for the postoperative aftercare of septic arthritis of the knee based on pathophysiological findings. Despite of a careful search of all internationally available arthroscopic pumps and devices, we were not able to locate any such device on the market.

### Accepted therapy

Clinical and experimental studies have shown a clear relationship between early and aggressive initiation of surgical treatment and success of therapy in septic arthritis of the knee [3-6,10-17]. If an infected knee joint is diagnosed early, and appropriate antibiotic therapy is initiated immediately, arthrotomy and needle aspiration can effectively eradicate the infection with only minimum damage to the cartilage [18]. Despite the positive results reported for repeated needle aspiration or tidal irrigation [18-20], sufficient drainage of the knee joint by these methods may be suboptimal [5,6,21]. Before the introduction of arthroscopy for the treatment of septic arthritis, open surgery with an early synovectomy was recommended [9,22-24]. The recommendations for postoperative aftercare, such as immobilization of the knee joint for 1 to 3 weeks versus immediate continuous passive motion (CPM) have already been discussed in the literature [9,23-27]. Salter et al. demonstrated that joint immobilization might lead to cartilage dystrophy via permanent localized pressure [26], with CPM therapy later being recommended [6,25,28,29]. Another controversial tool for postoperative aftercare, especially after open arthrotomy, was continuous suction irrigation therapy, which was based on the findings of Willenegger [30]. The aim was to wash out the joint and gain a dilution of aggressive proteolytic enzymes and reduction of the organisms. Turcic et al. demonstrated less effectiveness of conventional drain placement using a mathematical stream model compared to increased distance of the drains and the use of t-drains [31]. The main disadvantage of the continuous suction irrigation is the development of the so-called highway effect [5,10,14]. By taking the path of least resistance, the irrigation fluid flows through the joint without necessarily reaching all joint compartments. Jackson [11] and Jackson and Parsons [32] proposed a distension-irrigation technique in which the surgeon first irrigates and debrides the joint, then inserts two drains into the joint, distending the joint through the drains with saline solution, including antibiotic and mucolytic agents added over 3 hours, finally draining the joint for one more hour. In addition to the local distension-irrigation process, intravenous antibiotics are also administered. Some authors have recommended [33,34] or rejected [35,36] the use of continuous suction irrigation drains with antibiotics added to the irrigation solution. However, conclusive data have shown that antibiotic concentration in joint fluid can be achieved in adequate amounts after systemic use, leading the majority of authors not to add antibiotics into the irrigation solution [4,6,37-40].

In the 1980s arthroscopic management for septic knee arthritis was introduced. The authors achieved good results with arthroscopic treatment, and some authors used stages of joint infection based on pathophysiological findings (see table 1 for staging of Gächter and Jensen) or clinical appearance (see table 1 for staging of Kuner) with the purpose of recommending stage-dependent surgical therapy [5,6,10,11,14,25,28,29,33,35,37,41-43]. According to the literature the staging, as described by Gächter, was the most frequently used [4,6,37,42,44]. Studies could show that for stages Gächter I-III arthroscopic joint decompression with irrigation and debridement is effective. This may be repeated, if the septic process persists with further systemic antibiotic therapy [6,28,37]. This rate of repeated arthroscopic joint surgery was 0-41% and depended on the initial stage of the infection and time lapse between surgery and the onset of first infection signs [4,6,28,29,35,37,45,46]. Open surgery with arthrotomy is needed for stage Gächter IV or in cases of persisting infections after repeated arthroscopic joint surgery [6,37]. This occurred in 0-10% of the cases [4,6,28,29,35,37,45]. Late recurrences of joint infection after an asymptomatic interval are rare but can occur in 10% of the cases [4,6,28,29,35,37,45]. The overall healing rate of arthroscopic therapy in treating knee joint infections was high to a value of 90-100% [4,6,28,29,35,37,45,47-49].

**Table 1:** Stages of joint infection [5]

Gächter	The classification according to Gächter included IV Stages:
Stage I	Opacity of fluid, redness of the synovial membrane, possible petechial bleeding, no radiological alterations.

Stage II	Severe inflammation, fibrinous deposition, pus, no radiologic alterations.

Stage III	Thickening of the synovial membrane, compartment formation ("sponge-like" arthroscopic view, especially in the suprapatellar pouch), no radiologic alterations.

Stage IV	Aggressive pannus with infiltration of the cartilage, possibly undermining the cartilage, radiological signs of subchondral osteolysis, possible osseous erosions and cysts.

**Jensen**	**The classification according to Jensen included III stages:**

Stage I	Opaque effusion with high cell count, hyperaemia of the synovium.

Stage II	Putrid effusion, fibrin coatings, synovial hypertrophy with petechial bleeding.

Stage III	Severe villous synovitis with (partial) tamponade of the joint, synovial necrosis, synovial adhesions, cartilage destruction.

**Kuner**	**The classification according to Kuner was more a clinical description rather than an arthroscopic staging:**

Stage I	Purulent synovitis

Stage II	Joint empyema

Stage III	Panarthritis

Stage IV	Chronic arthritis.

The surgical treatment protocol for arthroscopic therapy of septic arthritis is joint decompression, elimination of the causative organisms by intensive lavage of the joint (with a minimum of 10 litres) with simultaneous elimination of proteolytic and lysosomal enzymes as well as toxins [1,4-7,10,11,28,35,37,41,45,47-50]. Debridement of necrotic soft tissues with the aim of preserving the synovial membrane as an immune competent structure and natural barrier, is recommended by the majority of the authors [4-6,10,25,28,29,33,35,37,44,47-49]. There is general consent for the use of culture-specific antibiotic therapy for about four to eight weeks. The protective effect of CPM should be used in postoperative aftercare [4,25-28,49].

Interestingly, some authors have recommended the use of continuous irrigation-suction drains after arthroscopic joint lavage [4,25,29,33], while others have recommended the so-called distension-irrigation described by Jackson [11,32,49]. Riel et al. could demonstrate a positive effect of this type of irrigation during the aftercare of arthroscopic treatment and concluded that further arthroscopic joint lavage could be reduced while conceding that their sample size was small and that further trials are needed [49]. The main weakness of the data concerning the aftercare with different irrigation modalities is the fact that the treated patient groups were very heterogeneous in terms of infection, etiology, infection staging, and postoperative irrigation modalities [4,6,11,25,29,32,33,49].

## Methods

### Novel pressure- and flow-controlled irrigation-suction system

The first step of the accepted therapy in septic arthritis is arthroscopic surgery with joint decompression, debridement, and joint irrigation following the detailed recommendations as described above. To optimize atraumatic joint decontamination, we suggest an efficient intra-articular irrigation-suction for the postoperative treatment phase using drains, which have been positioned during arthroscopy.

**Figure 1:**
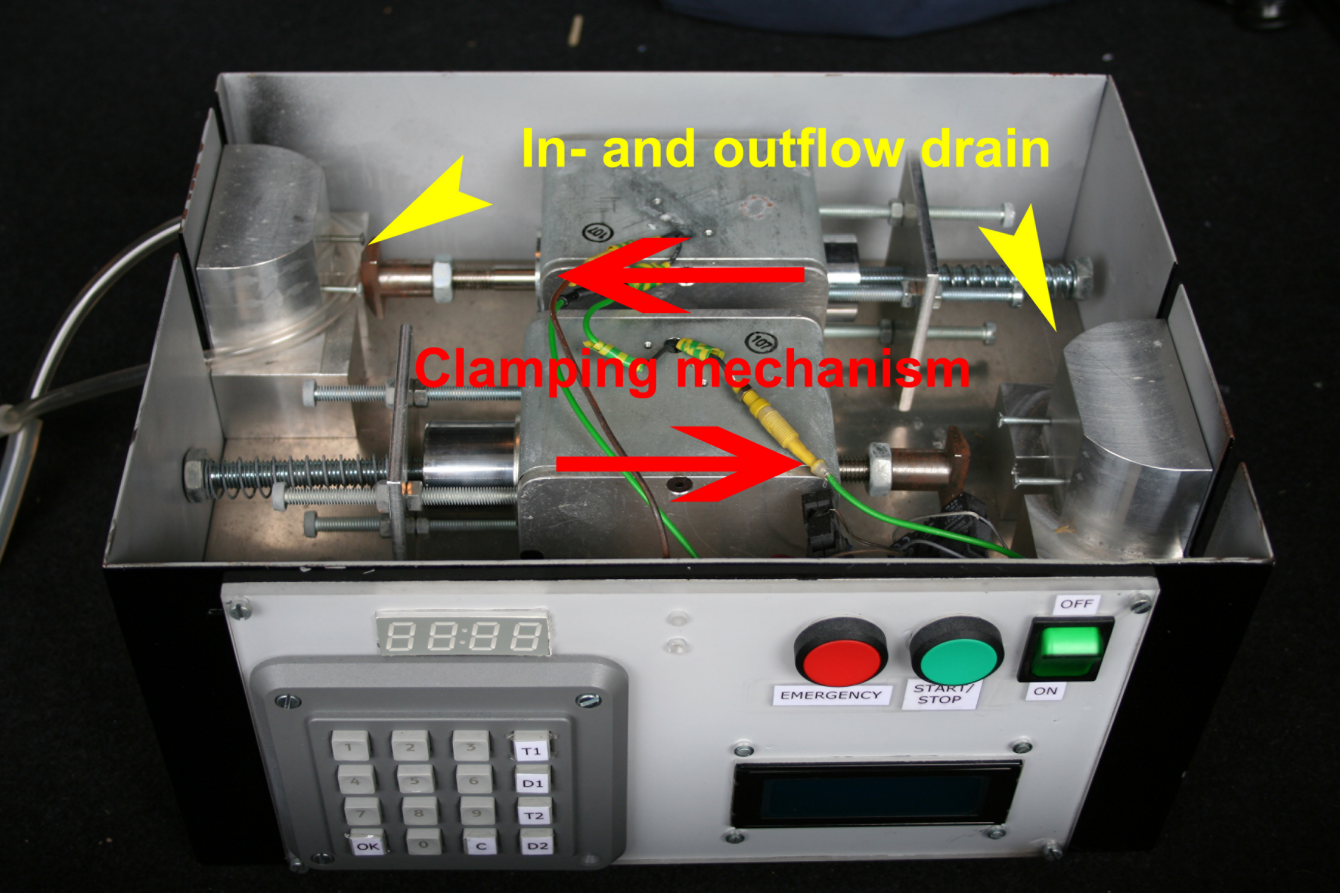
**Prototype coordinating the function of the arthroscopy, and suction pump**.

**Figure 2:**
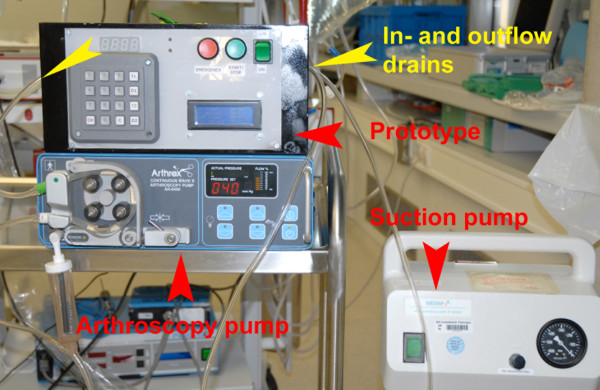
**Prototype, arthroscopy, and suction pump**.

The primary aim of our investigation was to develop a fully automatic device, which is much more efficient (defined by volume of irrigation per time) than the technique described by Jackson and Parsons [32], while applying, though, their principle. To achieve this, we needed an irrigation and suction pump. For the irrigating pump, we used a conventional arthroscopy pump from Arthrex^® ^providing pressure and flow-controlled conditions. For the suction pump, we used one from Medap^® ^with varying adjustable pressures (negative barometric pressures up to 600 mbar). The function of both pumps needed to be coordinated by a third device to achieve fully automatic irrigation and suction of the joint, while at the same time avoiding simultaneous suction and irrigation. We solved this requirement by developing a novel prototype, which independently clamps the in- and outflow drains. This prototype was developed in cooperation with a working group at a Technical University (Figure 1 and 2). To achieve maximum time flexibility of the irrigation and suction modalities, we defined and described the following four working steps with different time intervals, which can be chosen individually by the treating surgeon and supervised by the nursing staff and the patient:

T1 (time for irrigation in seconds): The joint is filled up and irrigated by the arthroscopic pump through the inflow drain and with defined pressure (e.g. between 20 and 80 mmHg) and flow (adjustable pumping volume per time). The arthroscopic pump keeps the joint distended (maintaining the chosen pressure) for the defined time period (T1) chosen by the surgeon. During this period the outflow drain is clamped in order that the suction pump remains idle.

D1 (Delay between irrigation and suction in seconds): Inflow and outflow drains are clamped simultaneously, while the joint is still distended. This time interval can be chosen by the surgeon.

T2 (Time for suction in seconds): The arthroscopic pump is idle by clamping the inflow drain. Suction is provided at this point by the de-clamped outflow drain. This time for suction can be adjusted by the surgeon individually.

D2 (Delay between suction and the onset of irrigation, end of one working circle): Now, both drains are clamped for a time period chosen by the surgeon, before the inflow drain is being de-clamped and another working cycle may begin.

The advantage of this automatic working system is that the surgeon is able to regulate the flow (volume per time) and the pressure for irrigation and suction. Painful joint distension can be reduced for short time periods or may be avoided by reducing the joint pressure due to the individual needs of the patient and pain situation. This problem was described by Jackson and Parsons [32]. Joint adhesions can be avoided by distending the capsule, providing simultaneously a very efficient postoperative joint decontamination, which is an important aspect of the pathophysiology and can be carried out using this additional tool for the postoperative aftercare of arthroscopy [4,29,32,49].

The prototype was equipped with an emergency stop button for the patient in case of a leakage into the soft tissue envelope with the danger of a compartment syndrome, if swelling or pain develops (Figure 1 and 2).

**Figure 3:**
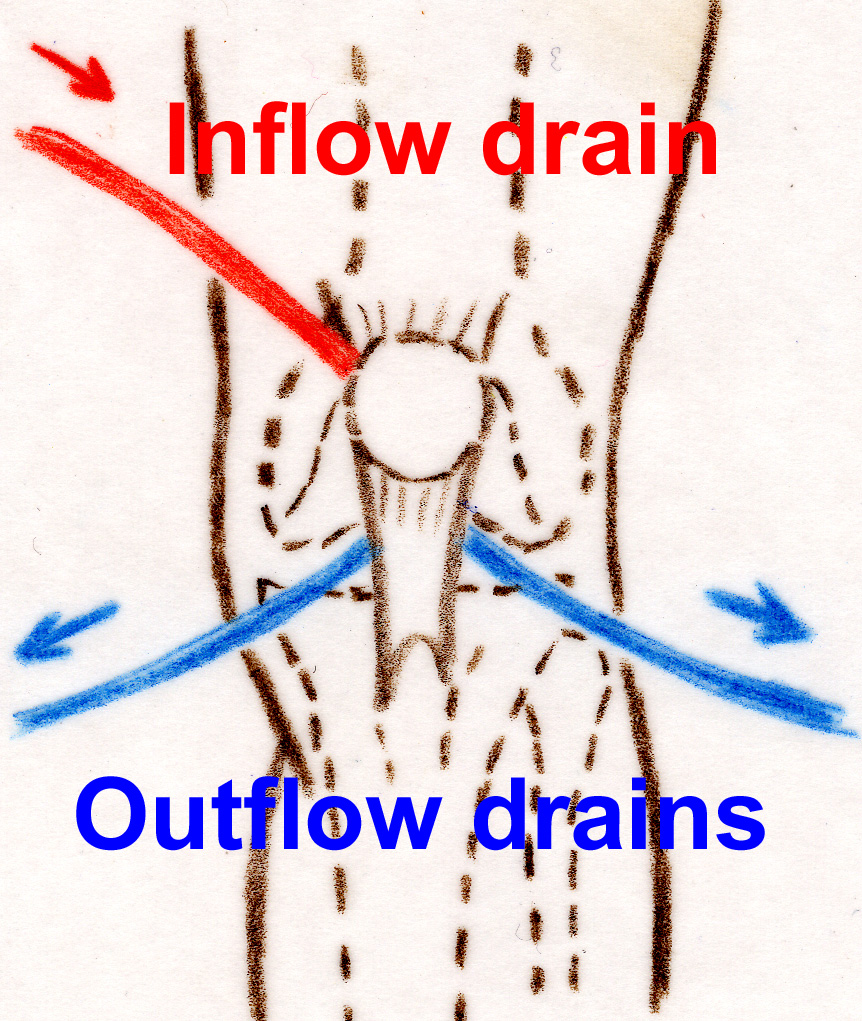
**Positioning of the drains in the knee**.

**Figure 4:**
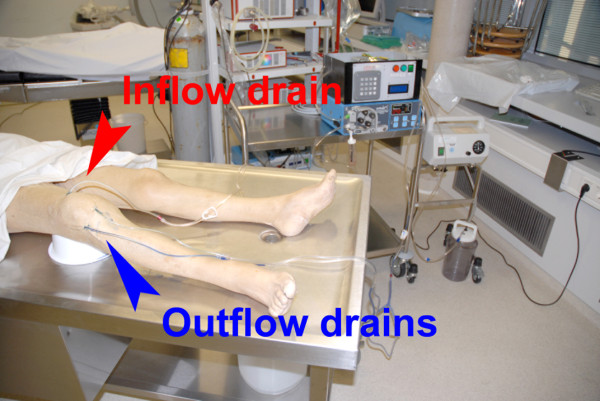
**Setting in the anatomic institute**.

We tested the effectiveness of treatment in a cadaveric study by comparing normal continuous suction irrigation versus the novel irrigation-suction system using our prototype in three cadavers. The knee joints were filled up with a volume of 80 ml methylene blue stain in formalin-fixed knee joints after three drains were placed arthroscopically. Two drains were placed antero-medially and laterally. The third drain was placed in the suprapatellar recessus for both irrigation systems (Figure 3 and 4). For both irrigation systems, the superior drain was used as the inflow tube and both anterior drains were used as outflow tubes. Simulation of postoperative irrigation thus began using physiologic saline solution.

The continuous suction irrigation system was characterized with the following parameters: The inflow tube was connected with conventional infusion systems using hydrostatic pressure (in a height of 1.8 m), while the outflow tubes were connected with the suction pump (Medap^®^), providing a continuous flow through the joint with a suction pressure of 300 mbar.

The novel irrigation system was characterized with the following parameters: The filling pressure was 40 mmHg (and high flow) with a suction pressure chosen at 300 mbar (T1 = 10 sec, D1 = 4 sec, T2 = 10 sec, D2 = 4 sec). To compare effectiveness we took a fluid specimen after 10 and 20 minutes and checked extinction via photometry at a wavelength of 500 nm. A line measure had previously been obtained by diluting the methylene blue stain in defined fractions. The extinction analysis was blinded.

This trial conforms to the Helsinki Declaration and to local legislation. The ethics committee of the Eberhard-Karls-University of Tübingen approved this trial.

## Results

The extinction measurements showed more effectiveness after 10 and 20 minutes for the novel irrigation-suction system. After ten minutes the average extinction was e_1C _= 0.8 for the continuous suction irrigation and e_1N _= 0.4 for the novel irrigation-suction system. After twenty minutes, we recorded an average extinction of e_2C _= 0.3 for continuous suction irrigation and e_2N _= 0.001 (macroscopic clear irrigation fluid) for the novel irrigation-suction system. As the irrigating solution was clear after twenty minutes, we stopped irrigation and performed an arthrotomy of all six knee joints. In the knee joints with continuous suction irrigation, methylene blue stain solution was visible in the postero-medial and lateral pouch and the posterior part of the notch, while clear solution was found in the knee joints irrigated by the novel irrigation-suction system. The students t-test showed significant differences, with a positive value for a confidence interval of 95% and a p < 0.001, demonstrating a superior clearance for the novel irrigation-suction.

## Discussion

Bacterial arthritis of the knee joint can be treated effectively by arthroscopy with positive results. Very important aspects of the pathophysiology and the consecutive therapy of joint infection are early joint decompression with joint irrigation and arthroscopic debridement.

Studies could show that for stages Gächter I-III arthroscopic joint decompression, irrigation and debridement is effective and can be repeated, if the infection persists [6,28,37]. The rate of repeated arthroscopy amounted to 0-41% and depended on the initial stage of the infection and time lapse between surgery and the onset of first infection signs [4,6,28,29,35,37,45,46]. Open surgery with arthrotomy is needed for stage Gächter IV joint infections or in cases of persisting infections after repeated arthroscopic joint surgery [6,37]. Late recurrences of joint infections after an asymptomatic interval are rare but can amount up to 10% [4,6,28,29,35,37,45]. The overall success and healing rate of arthroscopic therapy in treating knee joint infections was high (90-100%) [4,6,28,29,35,37,45,47-49].

Some authors rejected and others recommended the use of continuous irrigation-suction drains after arthroscopic joint irrigation [4,6,25,29,33], while others recommended the so-called distension-irrigation described by Jackson [18,32,49]. Riel et al. could demonstrate a positive effect in using this type of irrigation during the aftercare of arthroscopic surgery and concluded that further arthroscopic joint irrigation and the arthroscopic operating time could be reduced, while taking into account that their sample size was small and that further trials were needed [49]. The novel irrigation-suction system presented here provides an effective automatic joint irrigation regarding the irrigated fluid volume per time. This system has similar characteristics as the distension-irrigation described by Jackson and Parsons concerning the working cycle [32]. However, it has the great advantage of imitating arthroscopic irrigation characteristics. Regarding the components and the working cycle of this novel irrigation system, one will realize, that properties of the arthroscopic irrigation are provided and commenced during the postoperative aftercare. There are three main hypothesized advantages to continue an arthroscopic like irrigation in the postoperative phase: reducing the rate of repeated arthroscopic surgery and time for surgical irrigation, reducing joint adhesions with potential quicker recovery and better range of motion. We could not identify similar studies testing effectiveness of irrigating knee joints by different techniques, so that a further comparison and discussion of these results was limited. The mentioned hypothetic advantages of this novel irrigation system must be proven by further trials.

## Conclusions

This novel irrigation-suction system is more effective than conventional continuous suction irrigation and simultaneously avoiding highway effects by distending the joint capsule.

However, this system should be tested in cooperation with an industrial partner using different joints and further anatomic regions. The next steps would be to perform appropriate animal studies, the development and certification, and clinical tests such as a prospective randomized trial.

There seems to be a huge potential for the application of this novel device. Applications may include joint infections but also the postoperative care of other septic diseases such as bacterial peritonitis [50].

The value of an automated irrigation and suction device in the postoperative care is a new and promising technique, which will be evaluated by further studies.

## Competing interests

The authors declare that they have no competing interests.

## Authors' contributions

AA developed the idea of the novel irrigation system and carried out the requirements catalogue of the prototype and development with the technical university the prototype, performed the cadaver tests and drafted the manuscript. DA conceived of the cadaver test, and participated in its design and helped to draft the manuscript. SS participated in the design of the study and performed the statistical analysis. BH conceived and participated in the cadaver test. KW helped in drafting the manuscript and helped in filtering the relevant published data. JD gave intellectual input for the entire manuscript and drafting the manuscript. All authors read and approved the final manuscript.

## Pre-publication history

The pre-publication history for this paper can be accessed here:

http://www.biomedcentral.com/1471-2474/12/180/prepub
